# Cell-Free DNA Analysis of Fetal Aneuploidies in Early Pregnancy Loss

**DOI:** 10.3390/jcm13154283

**Published:** 2024-07-23

**Authors:** William H. Kutteh, Charles E. Miller, John K. Park, Victoria Corey, Mauro Chavez, Karen Racicot, Damian P. Alagia, Kristine N. Jinnett, Kirsten Curnow, Kristin Dalton, Sucheta Bhatt, David L. Keefe

**Affiliations:** 1 Department of Obstetrics & Gynecology, University of Tennessee Health Sciences Center and Baptist Hospital, Memphis, TN 38120, USA; 2 Recurrent Pregnancy Loss Center, Fertility Associates of Memphis, Memphis, TN 38120, USA; 3 Department of Clinical Sciences, Rosalind Franklin University of Medicine and Science, North Chicago, IL 60064, USA; chuckmillermd@gmail.com; 4 Carolina Conceptions, Raleigh, NC 27607, USA; john.park@carolinaconceptions.com; 5 Illumina, Inc., San Diego, CA 92122, USA; vcorey@illumina.com (V.C.); mchavez1@illumina.com (M.C.); kdalton@illumina.com (K.D.); sbhatt@illumina.com (S.B.); 6 Quest Diagnostics Inc., San Juan Capistrano, CA 92675, USA; karen.e.racicot@questdiagnostics.com (K.R.); damian.p.alagia@questdiagnostics.com (D.P.A.III); 7 Department of Obstetrics and Gynecology, NYU Langone Fertility Center, NYU Langone, New York, NY 10022, USA; david.keefe@umassmemorial.org

**Keywords:** cell-free DNA, miscarriage, early pregnancy loss, aneuploidy, noninvasive

## Abstract

**Background:** Products of conception samples are often collected and analyzed to try to determine the cause of an early pregnancy loss. However, sample collection may not always be possible, and maternal cell contamination and culture failure can affect the analysis. Cell-free DNA-based analysis of a blood sample could be used as an alternative method in early pregnancy loss cases to detect if aneuploidies were present in the fetus. **Methods**: In this prospective study, blood samples from early pregnancy loss patients were analyzed for the presence of fetal aneuploidies using a modified version of a noninvasive prenatal testing assay for cell-free DNA analysis. Results from cell-free DNA analysis were compared against the gold standard, microarray analysis of products of conception samples. This study was registered with ClinicalTrials.gov, identifier: NCT04935138. **Results**: Of the 76 patient samples included in the final study cohort, 11 were excluded from performance calculations. The 65 patient samples included in the final analysis included 49 with an abnormal microarray result and 16 with a normal microarray result. Based on results from these 65 samples, the study found that genome-wide cell-free DNA analysis had a sensitivity of 73.5% with a specificity of 100% for the detection of fetal aneuploidies in early pregnancy loss cases. **Conclusions**: This prospective study provides further support for the utility of cell-free DNA analysis in detecting fetal aneuploidies in early pregnancy loss cases. This approach could allow for a noninvasive method of investigating the etiology of miscarriages to be made available clinically.

## 1. Introduction

Early pregnancy loss (EPL), also known as miscarriage, occurs in about 10–20% of pregnancies and is estimated to affect one in four women who have been pregnant by the age of 39 [1,2]. Miscarriage is defined as the loss of a pregnancy before 20 weeks of gestation, although most occur within the first trimester [3]. Numerical chromosomal anomalies, including trisomies, monosomies, and sex chromosome abnormalities, cause about 50% of early pregnancy losses, with polyploidy, uniparental disomy, and copy-number variants (CNVs) accounting for around another 10% [4,5,6,7,8]. 

In cases of recurrent pregnancy loss (RPL, defined as ≥2 miscarriages) [9], recommended workups include the assessment of potential maternal etiologies, such as endocrine disease, autoimmune disease, and uterine abnormalities, as well as parental karyotype analysis [9,10]. This workup can be expensive and time-consuming, and it only identifies a probable cause for RPL in around half of patients [11]. Knowing the etiology of a miscarriage can be beneficial for future reproductive planning and management of subsequent pregnancies to help determine the risk for future pregnancy loss and the potential need for parental chromosome analysis and other workups [12,13], as well as providing psychological benefit to the patient and their partner [14,15].

To test for cytogenomic causes of pregnancy loss, products of conception (POC) may be surgically collected after the diagnosis of missed abortion. However, collection of a POC sample is not always possible, especially with the increasing number of medically managed miscarriages [16]. Moreover, maternal cell contamination (MCC) and/or culture failure can impact the success of POC analysis [3,17]. In recent years, studies have shown that cell-free (cf) DNA analysis may be used as a noninvasive means of detecting fetal aneuploidies in EPL cases [7,18,19,20,21,22]. Noninvasive prenatal testing (NIPT) using cfDNA has been in clinical use since 2011 and is a highly accurate and reliable method for detecting fetal chromosomal aneuploidies in pregnant patients [23]. A recent prospective cohort study showed that cfDNA-based testing in EPL patients is a robust method of evaluating fetal chromosomal status and could potentially improve the clinical management of these patients [7].

Our study objective was to use a modified version of a paired-end sequencing-based NIPT assay to screen for genome-wide fetal aneuploidies in patients with early miscarriage. This study also aimed to assess the performance of this assay by comparing the results of the EPL cohort against the gold standard, microarray analysis of POC samples. 

## 2. Materials and Methods

### 2.1. Study Design

In this prospective study, patients were recruited from six sites across the United States from June 2021 to December 2022. To be eligible for the study, participants had to be between 5 and 20 weeks of gestation with the miscarriage of a singleton pregnancy diagnosed by ultrasound. Additionally, pregnancy tissue had to be present in utero; patients with an empty sac were eligible for inclusion. Patients were excluded if they were <18 years of age, if there was no visible pregnancy tissue on the ultrasound, if the pregnancy had been conceived using in vitro fertilization with preimplantation testing for aneuploidy performed on the transferred embryo, if no microarray testing was planned on the POC sample, or if the patient received a normal result on either NIPT or diagnostic testing (chorionic villus sampling or amniocentesis) in the current pregnancy. Finally, if a patient was unable to provide consent, they were ineligible for the study. The study and all sites were approved by WCG^®^ IRB (#20204435), and the study was registered with ClincalTrials.gov (NCT04935138).

### 2.2. Sample Collection

Study participants underwent venipuncture to collect blood for cfDNA analysis (≥7 mL in a Streck tube) and MCC analysis (5 mL in an EDTA tube). Blood sample collection occurred after the patient had signed the consent form but prior to undergoing a surgical evacuation process. Following surgical evacuation, a 2 × 3 mm POC tissue sample was collected and sent to Quest Diagnostics Laboratory. Study participants were assigned a unique study ID number, and limited clinical information was collected for each patient on a case report form using this study ID number and the patient’s protected health information (date of birth) in a Health Insurance Portability and Accountability Act (HIPAA)-compliant manner. This included demographic information such as maternal age, gestational age (GA), current miscarriage history, and previous pregnancy/miscarriage history. In this study, RPL patients were defined as patients with ≥1 previous miscarriage who were undergoing their second or later pregnancy loss as part of this study. This was based on the American Society for Reproductive Medicine’s definition of RPL as ≥2 failed clinical pregnancies [9]. The expected clinical GA at sample collection (both POC and blood specimen collection) was based on information provided on the case report form, with dating determined by a variety of methods, including last menstrual period, first dating ultrasound, embryo transfer date, or date of intrauterine insemination. Sonographic GA at the time of ultrasound confirmation of miscarriage and the estimated time from fetal demise at the time of ultrasound confirmation of miscarriage were also collected. All patient data, including microarray results, were prospectively collected, de-identified, and stored in REDCap (Version 14.0.1), a HIPAA-compliant and secure web-based software platform designed to support data capture for research studies [24]. Results from the microarray and MCC analyses on POC specimens were reported back to the ordering clinician; results from the cfDNA analysis were not given to clinicians or their patients. 

### 2.3. Chromosomal Microarray Analysis of POC Specimens

POC specimen analysis was carried out by Quest Diagnostics Laboratory. The chromosomal microarray ClariSure Oligo-SNP was carried out on cultured chorionic villus tissue collected from POC samples to determine the presence of cytogenomic anomalies. This oligo-SNP (oligonucleotide, single nucleotide polymorphism, Affymetrix CytoScan HD) assay uses a microarray containing over 2.67 million probes, including 1.9 million copy-number probes and 750 thousand SNP probes.

### 2.4. Maternal Cell Contamination Analysis

Comparative analysis of maternal and fetal DNA for the presence of 15 short tandem repeats (CSF1PO, D2S1338, D3S1358, D5S818, D7S820, D8S1179, D13S317, D16S539, D18S51, D19S433, D21S11, FGA, THO1, TPOX, and vWA) to evaluate the presence of MCC was carried out by Quest Diagnostics Laboratory using multiplex PCR and capillary electrophoresis. This assay can detect a minor DNA species that is 5% or higher in a mixture of two DNA samples [25]. 

### 2.5. Cell-Free DNA Analysis

The cfDNA analysis was carried out by Illumina, Inc. using an adapted version of the VeriSeq™ NIPT Solution v2 assay (Illumina, Inc. Clinical Services Laboratory, Foster City, CA, USA) within 5 days of sample collection [26]. Briefly, plasma was isolated from patient whole blood samples, and cfDNA was extracted from the plasma for library preparation. Libraries were then quantified and pooled before undergoing paired-end whole-genome sequencing. As the goal was to analyze EPL samples at 24-plex, each sample was run in duplicate at 48-plex. Sequence data for each duplicate were combined after alignment to mimic sequencing depth at 24-plex, allowing for an average of 16 million reads. These merged data were then processed by VeriSeq NIPT Assay Software v2, with subsequent bioinformatics analysis and QC evaluation. Pregnancy loss-specific log-likelihood ratio (LLR) thresholds were established for the cfDNA assay as the intended use for LLR score cutoffs differs between EPL and NIPT samples. The LLR is the underlying numerical value or score per chromosome used for aneuploidy classification [26]. As EPL samples, on average, have an earlier gestational age and thus a lower fetal fraction, as well as a higher incidence of chromosomal aneuploidies than NIPT samples, thresholds need to be adjusted.

The cfDNA and POC microarray analyses were interpreted independently and compared at the time of threshold setting and downstream data analysis. EPL cfDNA results were compared to POC microarray results in the training set, and new thresholds were assessed. Due to the known limitations of the cfDNA testing platform, samples with POC array results indicating mosaicism, partial deletions or duplications less than 7 Mb, or triploidy, as well as samples with complete MCC and a normal female array result, were excluded from threshold selection and performance calculations. As we had a limited number of samples, all remaining samples were used for the training set and the performance set. The training set operated on the trisomy and monosomy LLR scores for each chromosome across all samples at once to set the trisomy and monosomy thresholds. The performance set operated by assigning classifications at the sample level to determine either full or partial concordance between the cfDNA results and POC microarray results.

EPL LLR monosomy and trisomy thresholds were defined using the training set with the interplay of sensitivity and specificity via receiver operating characteristic (ROC) curve analysis. In the ROC analysis for threshold determination, all chromosomes were treated as independent events with specificity defined by unaffected chromosomes (n = 1378) and sensitivity defined by affected chromosomes (n = 52, including 51 trisomies and one monosomy). Thresholds were selected that maximized sensitivity while maintaining 100% specificity in both trisomic and monosomic events. The EPL algorithm for sex chromosome classification was similar to that used for the VeriSeq NIPT Solution v2 [26]. Classification criteria for XO, XXX, and XXY abnormalities were re-established to achieve improved sensitivity and specificity relative to the NIPT thresholds against microarray-reported sex chromosomes.

Fetal fraction (FF) was estimated for all samples to determine the proportion of DNA originating from the cytotrophoblast that was present in the cfDNA sample. An FF estimation was provided for each sample based on both fragment size and coverage information [26]. 

## 3. Results

### 3.1. Sample Details

Of the 78 patients recruited for this study, one was excluded due to sample hemolysis, and another was excluded as the sample for cfDNA testing was not received by the Illumina laboratory. The final cohort that underwent cfDNA analysis (n = 76) had a median maternal age of 35.0 years, a median BMI of 27.7, and a median expected clinical GA at the time of sample collection of 8.7 weeks; see Table 1 for patient demographics. 

**Figure 1:**
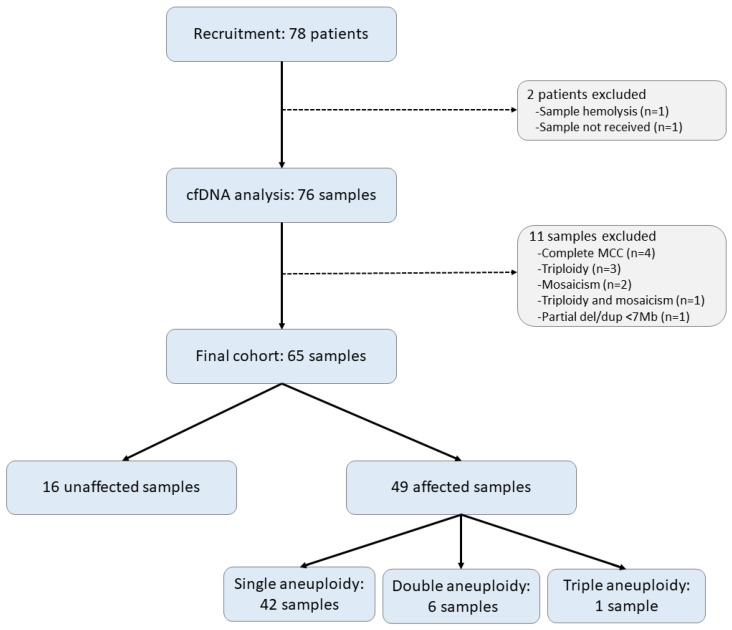
Sample flowchart. Samples were defined as affected or unaffected based on the presence or absence of chromosomal aneuploidy on the products of conception (POC) microarray. Abbreviations: MCC, maternal cell contamination; del/dup, deletion or duplication.

Eleven samples were excluded from LLR threshold selection and performance calculations, as outlined in Supplementary Section S1 and Table S1. Of the 65 samples included in the final analysis, 49 (75%) samples had an abnormal microarray result, including 42 with one aneuploidy, 6 with two aneuploidies, and 1 with three aneuploidies (Figure 1).

**Figure 2:**
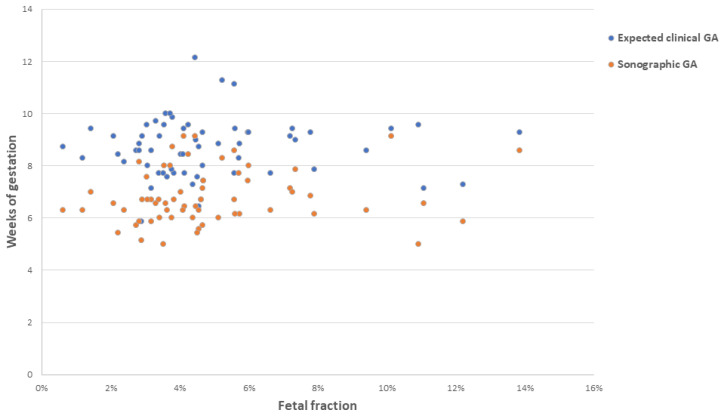
Relationship between expected clinical gestational age (GA), sonographic GA, and fetal fraction (FF) (n = 65). Expected clinical GA at the time of sample collection is represented by blue dots; sonographic GA at the time of the ultrasound confirmation of miscarriage is represented by orange dots.

For the final cohort (n = 65), the mean FF was 4.9%, with a median of 4.2% and a range of 0.6–13.9% (Figure 2). FF ranges were also evaluated for true positive, true negative, and discordant results, with no significant differences noted (Tables S2–S4).

**Figure 3:**
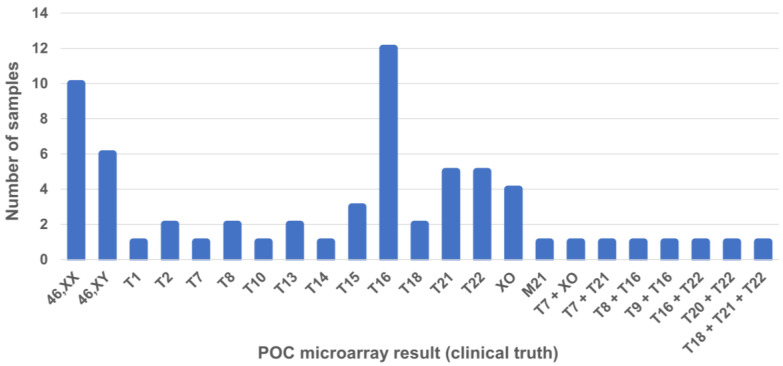
Products of conception (POC) microarray results for the samples used for algorithm threshold setting and performance assessment calculations (n = 65). Abbreviations: M, monosomy; T, trisomy.

All affected samples in the final analysis had whole chromosomal aneuploidies only. As shown in Figure 3, trisomy 16 was the most common single aneuploidy (n = 12) detected by the microarray analysis of POC samples, followed by trisomy 21 (n = 5) and trisomy 22 (n = 5). 

### 3.2. Training of the EPL Algorithm

As described above, thresholds were calculated from all EPL samples using a training set of all autosome monosomy and trisomy LLR scores with the corresponding POC microarray reported status (affected or unaffected). ROC analysis for trisomy classification resulted in a threshold of 3.4, which achieved a sensitivity of 70.6% in 51 positives for trisomy and a specificity of 100% in 1379 negatives in the training set (including all unaffected chromosomes and the one monosomy). ROC analysis for monosomy classification resulted in a threshold of 6.1, which achieved a sensitivity of 0% in one positive and a specificity of 100% in 1429 negatives (including all unaffected chromosomes and the 51 trisomies; Figure S1). Sex chromosome detection parameters were optimized to correctly classify 64 of the 65 samples in the training set, with one monosomy X (XO) case being called XX by the EPL algorithm. 

### 3.3. Performance of the EPL Algorithm

The performance of the EPL algorithm was determined by comparing the cfDNA results to the gold standard using POC microarray results. When assessing performance based on the full concordance approach, i.e., where each affected chromosome in an affected sample has to be correctly classified, we found a sensitivity of 69.4% (34/49) with a specificity of 100% (Table 2). The 34 samples correctly classified as true positives included 31 with one aneuploidy, 2 with two aneuploidies, and 1 with three aneuploidies (Table S2). When assessing the performance based on the partial concordance approach, i.e., where at least one affected chromosome in an affected sample was correctly classified, the sensitivity increased to 73.5% while maintaining 100% specificity (Table 2).

Thirteen cfDNA results were fully discordant with the microarray results (Table S3). The 16 cfDNA results with normal microarray results were correctly classified as true negatives by the EPL algorithm (Table S4).

## 4. Discussion

In this study, genome-wide cfDNA analysis of a blood sample from individuals experiencing an early pregnancy loss was able to determine the presence of fetal aneuploidies with 100% specificity and 73.5% sensitivity when using specific EPL LLR thresholds. Other studies noted sensitivities of ≥79% and specificities of ≥93% [7,21,22]. One study noted that the use of standard NIPT LLR thresholds in their cfDNA assay resulted in a sensitivity of 55% and a specificity of 100%, whilst the use of pregnancy loss-specific LLR thresholds gave a sensitivity of 82% and a specificity of 90% [18]. We targeted a high specificity in our study to avoid false-positive results. In testing alternate thresholds from ROC analyses, we found that sensitivity in the performance set failed to improve until specificity dropped from 100% to 81% due to limitations in sample size. Based on these analyses, we selected the highest specificity of 100%, which resulted in a sensitivity of 73.5%.

Overall, a 75% aneuploidy rate was identified by microarray in our POC samples, which is higher than generally reported for EPL cases [4,5]. This is likely reflective of the patient population in our study cohort, as samples were provided by reproductive endocrinology/infertility facilities, and many of these patients are expected to have a higher *a priori* fetal aneuploidy risk [27]. In our cohort, trisomy 16 was the most common aneuploidy, followed by trisomy 21 and trisomy 22. This was expected as trisomy 16 is thought to be the most common trisomy in pregnancies and is associated with a high probability of fetal death [28,29]. Similarly, trisomy 16 was the most commonly detected trisomy in previous studies looking at the use of cfDNA in pregnancy loss [7,18]. Trisomy 16 was also shown to be the most common aneuploidy in a large cohort of POC samples from EPL patients, followed by trisomy 21 and trisomy 22 [30].

For patients who experience an early pregnancy loss, obtaining a POC sample for analysis can be challenging for several reasons, including the increasing number of medically managed miscarriages. In addition, the success rate of POC analysis can be impacted by the presence of MCC and culture failure, depending on the testing methodology used [3,17]. Therefore, the use of a noninvasive method for determining the presence of a fetal aneuploidy could be a valuable additional or alternative approach for these patients and their providers. In our study, importantly, cfDNA analysis was able to identify a trisomy in one sample that failed to provide a result upon POC analysis due to complete MCC, demonstrating the value of cfDNA in potentially being able to successfully detect aneuploidy in a case that would have otherwise gone unresolved.

As shown here and in other studies, cfDNA analysis can be used to provide results in EPL cases at very early gestational ages. Despite the early GAs and low FFs in our study cohort, none of our samples failed to give a result. This is likely because the cfDNA assay used in this study did not use a fixed FF threshold for sample analysis but rather used a dynamic threshold for sample calling based on the FF of the sample and sequencing coverage, allowing samples to be called at very low FFs. Similarly, another study [18] found that cfDNA testing provided results before 8 weeks of gestation and at low FFs. The study by Schlaikjær Hartwig et al. [7] also noted that it was possible to obtain a result on cfDNA testing from 5 weeks of gestation.

Determining the reason why a miscarriage occurred is clinically important for several reasons, including providing psychological benefits to patients and their families. Studies have shown that both patients and their partners desire accurate and reliable information regarding the cause of their miscarriage [15,31]. The significant psychological morbidity affecting many patients following EPL is well described, with this psychological impact having possible long-term impacts on individuals and societies globally [14,32]. Of note, patients who receive information about the etiology of their loss report less distress, including reduced feelings of self-blame, guilt, grief, and anxiety [15,33,34]. As Zayyad et al. concluded, this distress could be reduced while also improving the overall well-being of patients and positively impacting their care by using cfDNA to identify a chromosomal abnormality as the cause of a miscarriage [35]. This information can also be used to estimate the recurrence risk and provide appropriate management for future pregnancies. 

A limitation of our study was the small study cohort, which did not allow for an extensive distribution of different chromosome anomalies. A larger data set is needed to refine the algorithm, improve the detection of some chromosomal aneuploidies, and address generalizations to larger, more diverse populations. There were also some technical limitations, namely that the cfDNA analysis platform and software used in this study could not detect triploidy or partial deletions/duplications < 7 Mb and may not have detected mosaicism. Finally, this cfDNA approach can only be used in a select group of patients who undergo a miscarriage, i.e., cases of missed abortion that are identified prior to the complete expulsion of POC. This is because the reported half-life of cfDNA is very short and disappears from maternal circulation within hours of POC expulsion [36]. Therefore, the POC tissue needs to be present when the blood sample is obtained to allow the cfDNA analysis to be carried out. Areas for future research include investigating the time limitations of when a blood sample can be drawn for cfDNA analysis in cases of missed abortion to see if it is possible to obtain an informative cfDNA result from blood drawn shortly after POC expulsion and further assessing the performance of cfDNA analysis based on the gestational age at which the early pregnancy loss occurs.

## 5. Conclusions

This study provides further support regarding the ability of a cfDNA approach to screen for fetal chromosomal abnormalities in EPL cases, allowing for a noninvasive method of investigating the etiology of miscarriages to be made available clinically. This could help inform the medical management of these patients, with the goal of improving reproductive planning and outcomes for individuals and couples impacted by pregnancy loss. 

## Figures and Tables

**Table 1:** Demographics of study participants.

Variable	Study Cohort (Total n = 76)	Final Analysis Samples (Total n = 65)
**Maternal age, yr ^a^**		
Mean	34.9	34.9
Median	35.0	35.0
Range	25.0–44.0	25.0–44.0
**Expected clinical GA at time of sample collection, wk ^b^**		
Mean	8.7	8.7
Median	8.7	8.7
Range	5.9–12.1	5.9–12.1
**Sonographic GA at time of** **ultrasound confirmation of** **miscarriage, wk**		
Mean	6.7	6.8
Median	6.4	6.6
Range	5.0–9.1	5.0–9.1
**BMI**		
Mean	29.8	29.7
Median	27.7	27.5
Range	18.1–47.8	18.1–47.8
**Previous pregnancies, n (%) ^c^**		
0	19 (25)	16 (25)
1	20 (26)	17 (26)
2	16 (21)	14 (22)
≥3	21 (28)	18 (28)
**Previous miscarriages, n (%)**		
0	41 (54)	34 (52)
1	18 (24)	17 (26)
2	5 (7)	4 (6)
3	7 (9)	6 (9)
4	3 (4)	2 (3)
5	1 (1)	1 (2)
6	1 (1)	1 (2)
**Previous pregnancy with a chromosomal abnormality, n (%) ^d^**		
Yes	8 (11)	8 (12)
No	68 (90)	57 (88)
**Use of ART in current** **pregnancy, n (%)**		
Yes	37 (49)	30 (46)
No	39 (51)	35 (54)

^a^ Includes one case of a pregnancy conceived via egg donor, and for this case, the egg donor’s age (31 years) was used for the maternal age. ^b^ Expected clinical gestational age (GA) at the time of sample collection was unknown for three patients. ^c^ Not dependent on whether the pregnancy resulted in the birth of a child. ^d^ Based on patient-reported information without medical verification. Abbreviations: ART, assisted reproductive technology; BMI, body mass index; n, number; wk, weeks; and yr, years.

**Table 2:** Performance of the EPL algorithm in the detection of fetal aneuploidies.

Sample Level Calculations	Sensitivity (%)	Specificity (%)
Full concordance ^a^	69.4	100
Partial concordance ^b^	73.5	100

Performance was based on a comparison of the gold standard determined by POC microarray results to cfDNA results. ^a^ All aneuploidies identified by a microarray on POC were also identified by cfDNA analysis. ^b^ At least one of the aneuploidies identified by a microarray on POC was also identified by cfDNA analysis. Abbreviations: cfDNA, cell-free DNA; EPL, early pregnancy loss; and POC, products of conception.

## Data Availability

All de-identified data used for this study are included in the main text and Supplementary Materials. Case-level data are not available due to legal and ethical requirements to protect patient privacy.

## Supplementary Materials

The following supporting information can be downloaded at https://www.mdpi.com/article/10.3390/jcm13154283/s1. Section S1: Description of 11 samples excluded from performance calculations; Table S1: Details of the 11 samples excluded from performance calculations; Table S2: Details of the 34 fully concordant and 2 partially concordant calls by the EPL algorithm; Table S3: Details of the 13 discordant calls by the EPL algorithm; Table S4: Details of the 16 true-negative calls by the EPL algorithm; Figure S1: Autosome trisomy and monosomy log-likelihood ratio (LLR) threshold training.
