# Supplementary material for: Cell-Free DNA Analysis of Fetal Aneuploidies in Early Pregnancy Loss
Source: J Clin Med. 2024 Jul 23;13(15):4283. doi: 10.3390/jcm13154283 (PMC11313239; doi:10.3390/jcm13154283)

SUPPLEMENTARY MATERIALS

Cell-free DNA Analysis of Fetal Aneuploidies in Early Pregnancy Loss

William H. Kutteh[1,2,*], Charles E. Miller[3], John K. Park[4], Victoria Corey[5], Mauro Chavez[5], Karen Racicot[6], Damian P. Alagia III[6], Kristine N. Jinnett[5], Kirsten Curnow[5], Kristin Dalton[5], Sucheta Bhatt[5] and David L. Keefe[7]

[1]Department of Obstetrics & Gynecology, University of Tennessee Health Sciences Center and Baptist Hospital, Memphis, TN 38120, USA
[2]Recurrent Pregnancy Loss Center, Fertility Associates of Memphis, Memphis, TN 38120, USA
[3]Department of Clinical Sciences, Rosalind Franklin University of Medicine and Science, North Chicago, IL 60064, USA
[4]Carolina Conceptions, Raleigh, NC 27607, USA
[5]Illumina, Inc., San Diego, CA 92122, USA
[6]Quest Diagnostics Inc., San Juan Capistrano, CA 92675, USA
[7]Department of Obstetrics and Gynecology, NYU Langone Fertility Center, NYU Langone, New York, NY 10022, USA
*Correspondence: wkutteh50@gmail.com

Table of Contents

Section S1	Description of 11 samples excluded from performance calculations	2
Table S1	Details of the 11 samples excluded from performance calculations	3
Table S2	Details of the 34 fully concordant and 2 partially concordant calls by the EPL algorithm	4
Table S3	Details of the 13 discordant calls by the EPL algorithm	6
Table S4	Details of the 16 true-negative calls by the EPL algorithm	7
Figure S1	Autosome trisomy and monosomy log likelihood ratio (LLR) threshold training	8

Section S1

Description of 11 samples excluded from performance calculations.

Four samples were excluded from log likelihood ratio (LLR) threshold selection and performance calculations due to complete maternal cell contamination (MCC) in the products of conception (POC), with a normal female array result [arr(X,1-22)x2] in the POC, as clinical truth of the fetal chromosome status could not be determined (Table S1).

Seven additional samples were excluded from LLR threshold selection and performance calculations as the chromosomal abnormalities detected by POC microarray analysis are not expected to be detected by the cell-free (cf) DNA analysis due to limitations of the cfDNA testing methodology (POC array results with mosaicism, partial deletion or duplication <7Mb, and/or triploidy).

As shown in Table S1, cfDNA analysis identified trisomy 9 in one sample that failed to provide an informative POC microarray result due to complete MCC (sample #1). Also, for sample #11, where POC microarray yielded a complex result of arr(X)x2,(Y)x1,(1-6)x3,(7)x3~4,(8-12)x3,(13)x4,(14-22)x3 (near-triploidy with tetrasomy 13 and mosaic tetrasomy 7), the cfDNA result accurately detected an increased signal of chromosomes 7 and 13, reporting out a result of trisomy 7 and trisomy 13, although this was not concordant with the result by microarray.

See Table S1 for details of all excluded samples.

Table S1. Details of the 11 samples excluded from performance calculations.

Sample	Exclusion reason (based on POC array result)	POC microarray result	cfDNA result
1[a]	Complete MCC and arr(1-22,X)x2 array result	arr(1-22,X)x2[a]	AD, result consistent with trisomy 9 and XX
2[a]	Complete MCC and arr(1-22,X)x2 array result	arr(1-22,X)x2[a]	NAD, result consistent with XX
3[a]	Complete MCC and arr(1-22,X)x2 array result	arr(1-22,X)x2[a]	NAD, result consistent with XX
4[a]	Complete MCC and arr(1-22,X)x2 array result	arr(1-22,X)x2[a]	NAD, result consistent with XY
5[b]	Partial deletion or duplication <7Mb	arr[GRCh37] 18q22.1q22.3(65094544_69376308)x1, male	NAD, result consistent with XY
6[c]	Mosaicism	arr(12)x2-3, male	NAD, result consistent with XY
7[d]	Mosaicism	arr[GRCh37] 1q42.3q44(234851538_249224684)x1[0.32],(18)x1[0.35], male	NAD, result consistent with XY
8	Triploidy	arr(X,1-22)x3	NAD, result consistent with XX
9	Triploidy	arr(1-22)x3,(X)x2,(Y)x1	NAD, result consistent with XY
10	Triploidy	arr(1-22)x3,(X)x2,(Y)x1	NAD, result consistent with XY
11	Near-triploidy and mosaicism	arr(X)x2,(Y)x1,(1-6)x3,(7)x3-4,(8-12)x3,(13)x4,(14-22)x3[e]	AD, result consistent with trisomy 7 and trisomy 13 and XY

AD, aneuploidy detected; arr, array; cf, cell-free; MCC, maternal cell contamination; NAD, no aneuploidy detected; POC, products of conception.
[a]Uninformative due to complete maternal cell contamination.
[b]Approximately 4.3 Mb interstitial deletion of 18q22.1q22.3.
[c]No comment regarding level of mosaicism on array report.
[d]Mosaic monosomy 18 and mosaic terminal deletion of 1q42.3qter (approximately 14.4 Mb) in approximately 32–35% of cells.
[e]Near-triploidy, 70-71 chromosomes due to the presence of an extra haploid set of chromosomes, except tetrasomy for chromosome 13 and mosaic tetrasomy for chromosome 7.

Table S2. Details of the 34 fully concordant and 2 partially concordant calls by the EPL algorithm.

Sample	Gestational age, wk[a]	Fetal fraction, %	POC microarray result	cfDNA result
1	7.7	3.4	arr(7)x3, female	AD, result consistent with trisomy 7 and XX
2	7.7	4.1	arr(8)x3, male	AD, result consistent with trisomy 8 and XY
3	9.3	4.7	arr(8)x3, male	AD, result consistent with trisomy 8 and XY
4	8.6	9.4	arr(10)x3, male	AD, result consistent with trisomy 10 and XY
5	8.3	5.7	arr(13)x3, female	AD, result consistent with trisomy 13 and XX
6	9.1	7.2	arr(15)x3, male	AD, result consistent with trisomy 15 and XY
7	8.6	3.2	arr(15)x3, female	AD, result consistent with trisomy 15 and XX
8	9.4	5.6	arr(15)x3, female	AD, result consistent with trisomy 15 and XX
9	8.4	4.1	arr(16)x3, female	AD, result consistent with trisomy 16 and XX
10	Not available	3.2	arr(16)x3, female	AD, result consistent with trisomy 16 and XX
11	8.7	4.6	arr(16)x3, female	AD, result consistent with trisomy 16 and XX
12	7.9	7.9	arr(16)x3, female	AD, result consistent with trisomy 16 and XX
13	7.3	12.2	arr(16)x3, female	AD, result consistent with trisomy 16 and XX
14	7.3	4.4	arr(16)x3, male	AD, result consistent with trisomy 16 and XY
15	10.0	3.6	arr(16)x3, female	AD, result consistent with trisomy 16 and XX
16	9.7	3.3	arr(16)x3, female	AD, result consistent with trisomy 16 and XX
17	12.1	4.4	arr(18)x3, male	AD, result consistent with trisomy 18 and XY
18	10.0	3.7	arr(18)x3, male	AD, result consistent with trisomy 18 and XY

Sample	Gestational age, wk[a]	Fetal fraction, %	POC microarray result	cfDNA result
19	9.4	10.1	arr(21)x3, male	AD, result consistent with trisomy 21 and XY
20	9.0	7.4	arr(21)x3, female	AD, result consistent with trisomy 21 and XX
21	8.9	2.8	arr(21)x3, male	AD, result consistent with trisomy 21 and XY
22	7.4	4.7	arr(21)x3, male	AD, result consistent with trisomy 21 and XY
23	7.1	11.1	arr(21)x3, female	AD, result consistent with trisomy 21 and XX
24	7.7	5.6	arr(22)x3, female	AD, result consistent with trisomy 22 and XX
25	9.3	7.8	arr(22)x3, male	AD, result consistent with trisomy 22 and XY
26	9.6	4.2	arr(22)x3, male	AD, result consistent with trisomy 22 and XY
27	9.3	6.0	arr(22)x3, male	AD, result consistent with trisomy 22 and XY
28	9.3	6.0	arr(22)x3, male	AD, result consistent with trisomy 22 and XY
29	11.1	5.6	arr(X)x1	AD, result consistent with monosomy X
30	11.3	5.2	arr(X)x1	AD, result consistent with monosomy X
31	9.4	4.1	arr(X)x1	AD, result consistent with monosomy X
32	8.9	5.7	arr(X)x1,(7)x3	AD, result consistent with trisomy 7 and monosomy X
33	7.7	3.5	arr(8)x3,(16)x3, male	AD, result consistent with trisomy 8, trisomy 16, and XY
34	9.1	2.9	arr(18)x3,(21)x3,(22)x3, female	AD, result consistent with trisomy 18, trisomy 21, trisomy 22, and XX
35[b]	9.0	4.5	arr(16)x3,(22)x3, male	AD, result consistent with trisomy 16 and XY
36[b]	8.9	5.1	arr(20)x3,(22)x3, male	AD, result consistent with trisomy 20 and XY

AD, aneuploidy detected; arr, array; cf, cell-free; EPL, early pregnancy loss; POC, products of conception; wk, week.
[a]Expected clinical gestational age at time of sample collection.
[b]Partially concordant case, with one true-positive and one false-positive result.

Table S3. Details of the 13 discordant calls by the EPL algorithm.

Sample	Gestational age, wk[a]	Fetal fraction, %	POC microarray result	cfDNA result
1	5.9	2.9	arr(1)x3, female	NAD, result consistent with XX
2	7.9	3.8	arr(2)x3, male	NAD, result consistent with XY
3	9.6	3.0	arr(2)x3, female	NAD, result consistent with XX
4	9.1	2.1	arr(13)x3, female	NAD, result consistent with XX
5	8.7	0.6	arr(14)x3, male	NAD, result consistent with XY
6	7.1	3.2	arr(16)x3, male	NAD, result consistent with XY
7	8.6	2.8	arr(16)x3, female	NAD, result consistent with XX
8	8.6	2.7	arr(16)x3, male	NAD, result consistent with XY
9	9.1	3.4	arr(16)x3, female	NAD, result consistent with XX
10	6.7	4.6	arr(21)x1, male	NAD, result consistent with XY
11	8.0	3.1	arr(X)x1	NAD, result consistent with XX
12	6.4	4.6	arr(7)x3,(21)x3, male	NAD, result consistent with XY
13	7.6	4.5	arr(9)x3,(16)x3 male	NAD, result consistent with XY

arr, array; cfDNA, cell-free DNA; EPL, early pregnancy loss; NAD, no aneuploidy detected; POC, products of conception; wk, week.
[a]Expected clinical gestational age at time of sample collection.

Table S4. Details of the 16 true-negative calls by the EPL algorithm.

Sample	Gestational age, wk[a]	Fetal fraction, %	POC microarray result	cfDNA result
1	8.3	1.2	arr(X,1-22)x2	NAD, result consistent with XX
2	Not available	4.2	arr(X,1-22)x2	NAD, result consistent with XX
3	Not available	3.8	arr(X,1-22)x2	NAD, result consistent with XX
4	9.3	13.9	arr(X,1-22)x2	NAD, result consistent with XX
5	8.0	4.7	arr(X,1-22)x2	NAD, result consistent with XX
6	9.4	7.3	arr(X,1-22)x2	NAD, result consistent with XX
7	9.4	1.4	arr(X,1-22)x2	NAD, result consistent with XX
8	7.7	3.8	arr(X,1-22)x2	NAD, result consistent with XX
9	9.6	10.9	arr(X,1-22)x2	NAD, result consistent with XX
10	7.7	6.6	arr(X,1-22)x2	NAD, result consistent with XX
11	8.4	4.0	arr(1-22)x2(X,Y)x1	NAD, result consistent with XY
12	8.1	2.4	arr(1-22)x2(X,Y)x1	NAD, result consistent with XY
13	9.9	3.8	arr(1-22)x2(X,Y)x1	NAD, result consistent with XY
14[b]	9.6	3.5	arr[GRCh37] 1q25.3q31.3(182840619_197725208)x2 hmz, 7p12.3p12.1(47485965_52803539)x2 hmz, 7q22.3q31.1(106619472_111629746)x2 hmz, male	NAD, result consistent with XY
15	7.6	3.6	arr(1-22)x2(X,Y)x1	NAD, result consistent with XY
16	8.4	2.2	arr(1-22)x2(X,Y)x1	NAD, result consistent with XY

arr, array; cfDNA, cell-free DNA; EPL, early pregnancy loss; hmz, homozygosity; NAD, no aneuploidy detected; POC, products of conception; wk, week.
[a]Expected clinical gestational age at time of sample collection.
[b]No copy-number variations identified; three regions of homozygosity detected, combined length of regions of homozygosity was approximately 25.2 Mb.

Figure S1. Autosome trisomy and monosomy log likelihood ratio (LLR) threshold training. Trisomy and monosomy LLR thresholds were selected that resulted in 100% specificity in training data.

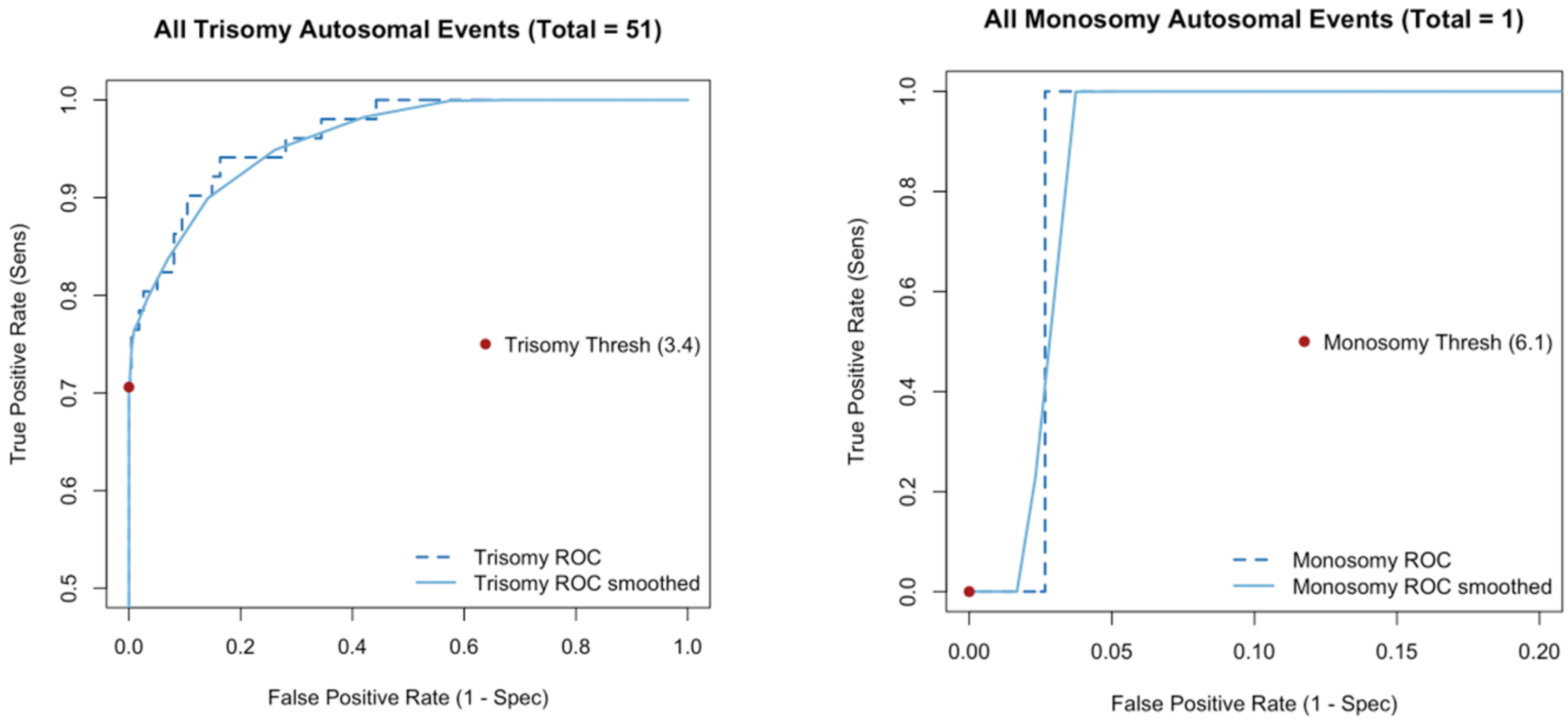

Supplement: Supplementary file 1 [file jcm-13-04283-s001.zip › jcm-3062011-supplementary.pdf]
